# Jewish Spaces in Present Vienna: A Relational, Hybrid Approach

**DOI:** 10.1007/s12397-024-09548-8

**Published:** 2024-07-02

**Authors:** Susanne Korbel

**Affiliations:** University of Graz, Graz, Austria

**Keywords:** Vienna, Relational space making, Jewish–non-Jewish relations, Urban studies, Virtual spaces

## Abstract

In October 2017, Vienna’s *Leopoldstadt* community succeeded in reinstalling a Hebrew street sign in a public space of the second district. This achievement became possible in large part due to the efforts of an active online community that encouraged many people to share their wish to have visible signs of the former historic Jewish quarter in the present urban space. Through vigorous Facebook and other social media activities, the interest that the group generated put pressure on the city, leading to the support of an art project. The placement of the Hebrew street sign marked a hybrid way of constructing Jewish urban spaces. The dialogue between virtual and physical spaces added new layers to the historic Jewish quarter of Vienna; this way of relational space making is, I wish to argue, paradigmatic for today’s Europe as it witnesses the heyday of Holocaust tourism and *klezmer* revivals. In this article, I investigate this space-making process in Jewish public history in present Vienna and examine how the virtual community frames the way urban Jewish spaces are constructed.

On building number 5 in Vienna’s *Taborstrasse*, close to *Karmeliterplatz*, not far from the Danube Canal, a Hebrew street sign has been visible since fall 2017. In this photograph (Fig. 1), the street sign “Tavorstraße” gleams in the sun, lending a Jewish flavor that spreads from one of the central streets throughout a district that is—today again—known as a Jewish neighborhood. The sign, an art installation that renders the name of the street in Hebrew (not Yiddish!)—a nod to the backdrop of the metropolis in itself—is modeled according to the standardized signs for street nomination in a public space and is placed where street signs are intended to be hung. This street sign, as it is to be seen again today, was relocated on 10 October 2017, some 30 m further toward the Danube than it was initially placed at Taborstraße 18. This relocation marked the end of an online debate and initiative that aimed to replace a Hebrew street sign that was irregularly installed and soon after removed in the part of the city renowned as a (former) Jewish space.

**Fig. 1:**
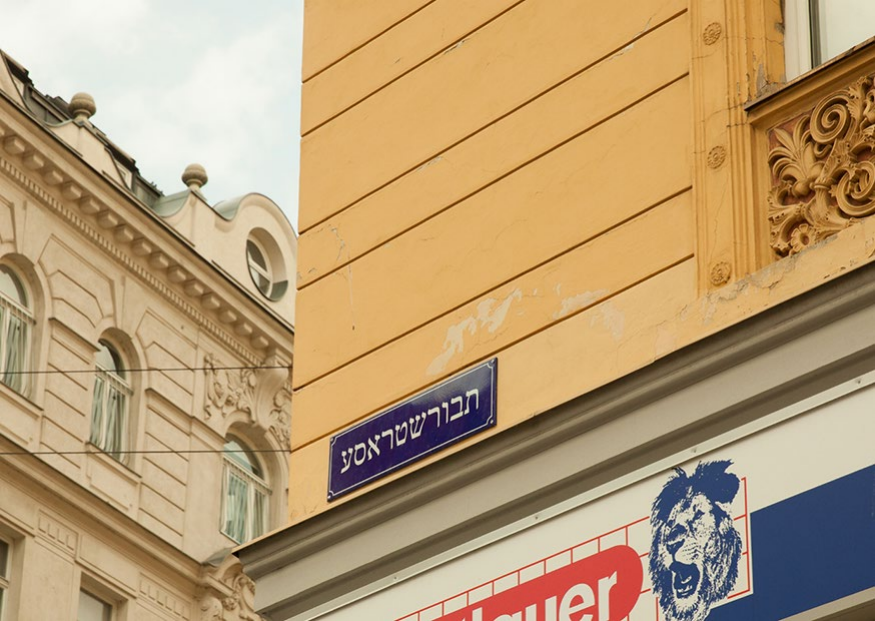
Hebrew street sign “Tavorstraße” at Taborstraße 5 in Vienna’s second district, © kindly provided by the artists Shabi Fiumei

In this article, I probe how contemporary Jewish urban spaces are designed, interpreted, and lived. As a case study, I use the installation of the Hebrew street sign at *Taborstrasse* 5/18 in Vienna’s second district, *Leopoldstadt*, in 2017. The installation was initially created on the initiative of the Jewish artist, who felt irritated by the presence of the memory of antisemitism and the absence of a reminder of the former living Jewish culture. When the municipal administration removed the unregistered sign, an online community managed to get it reinstated. In this example, a local and a virtual community, consisting of Jews and non-Jews alike, mutually constructed or added to an urban Jewish space. I thus ask how peoples’ contemporary perceptions of their cities influence Jewish spaces. What avenues of expression do current examples of space making include? How do new spatial practices influence present Jewish and non-Jewish cultures? First, I introduce my case study. To approach this example of contemporary Jewish space making, I then provide an overview on how spatial considerations found their way into Jewish studies and which research interests have been investigated in the course of this so-called Jewish spatial turn. This leads me to argue that the (Jewish) spatial turn is currently drifting toward the virtual sphere, also beyond a metaphorical sense, since increasingly relational processes of space making, which emerge from or include virtual spaces, occur.

## Virtual Activism for a Hebrew Street Sign in Vienna’s *Leopoldstadt*

On 10 October 2017, Vienna’s—Jewish and non-Jewish—*Leopoldstadt* community achieved their wish: the administration of the city of Vienna (re)installed a Hebrew street sign in the public space of the second district. This sign had initially been erected and quickly afterward removed the previous summer. But how did it happen that a Hebrew street sign became a focal point for negotiating public Jewish spaces?

To situate these events more adequately, I briefly touch upon the historical context: Vienna’s second district, also known as *Leopoldstadt*, was and is renowned as a “Jewish quarter” far beyond the city’s borders. It was the historical settlement area of the Jewish community in early modern times and at the turn of the twentieth century was home to many synagogues and Jewish institutions—today, it partially is again. The second district of Vienna functioned as a hub for both orthodox and ‘assimilated’ Jews for many reasons. For instance, the *Leopoldstadt* also hosted the northern railway station, which was, around 1900, the first port of call for Jewish immigrants from Eastern Europe. Additionally, Vienna’s largest synagogue, the *Leopoldstätter Tempel* (the Leopoldstätter Synagogue), was located close to Taborstraße there. It added past and present—today through a void and a memory plaque—to the Jewish history and culture of the district (Korbel 2021, 64). *Nestroyhof*, the building next to the synagogue, represented Jewish culture at the turn of the twentieth century and does so again today, hosting the theater *HaMakom—der Ort* (the place) (Hamakom 2023). It was this that earned the district the name *Mazzesinsel* and put it in the heart of modern tourist activities related to Jewish heritage and memory (Beckermann 1992).

It was there, in the *Taborstraße,* that an initially unknown person or group put up a street sign in Hebrew letters in late June 2017. The sign immediately caught the eye of passers-by, drew stares, and turned into the buzz of the district. Generally appreciated by the inhabitants of the neighborhood—Jewish and non-Jewish alike—discussions about the street sign and the Jewish past and present circulated through the district and were soon featured in local newspapers. It received attention, but it turned out that it had not been approved by the *Bezirksmagistrat* (district authority). Despite Austrian authorities usually trying (at least to pretend) to care for Jewish cultural sites and memory initiatives, the city administration “fulfilled their administrative duty” (this might remind the reader of a different historical debate in Austria post-Holocaust history) and removed the street sign on 17 July 2017 (“Rätsel” 2017c). The absence was noticed by residents, and local newspapers began to report on it.

Soon, the removal of the Hebrew street sign just a few weeks after its installation at Taborstraße 18 caused protest, performed on the Internet. The neighborhood formed a group that—to facilitate coordination and to achieve outreach and establish a network beyond the district and across the city of Vienna—chose to rely on a virtual presence (Facebook 2017b). Together with people participating online, this pop-up community created an online platform using social media (Facebook and Instagram) and promoted their wish to have “the Yiddish [!] street sign” back. Operating as the Facebook groups “Das Taborstraße-Schild auf Jiddisch soll zurück”^1^ and “Taborstraße Straßenschild/תבורשטראסע שלט רחוב,”^2^ they articulated a strong affiliation with the sign because it reminded the locals, according to their argumentation, of lived historic Jewish spaces, such as the Jewish entertainment mile that had crucially determined Jewish and non-Jewish everyday life in the neighborhood at the beginning of the twentieth century (Correspondence 2023). In the course of the online campaign, the community learned that the Hungarian Jewish street artist Sebestyén Fiumei (alias Shabi Fiumei)^3^ was the mastermind behind the installation and had initially placed it within view of a window of his apartment. “Since the house on Tabor Street has been standing there for years without a street sign for some reason, I thought I’d do it myself, add something to it, a little work of art so to speak” (Facebook Gruppe 2017b).

**Fig. 2:**
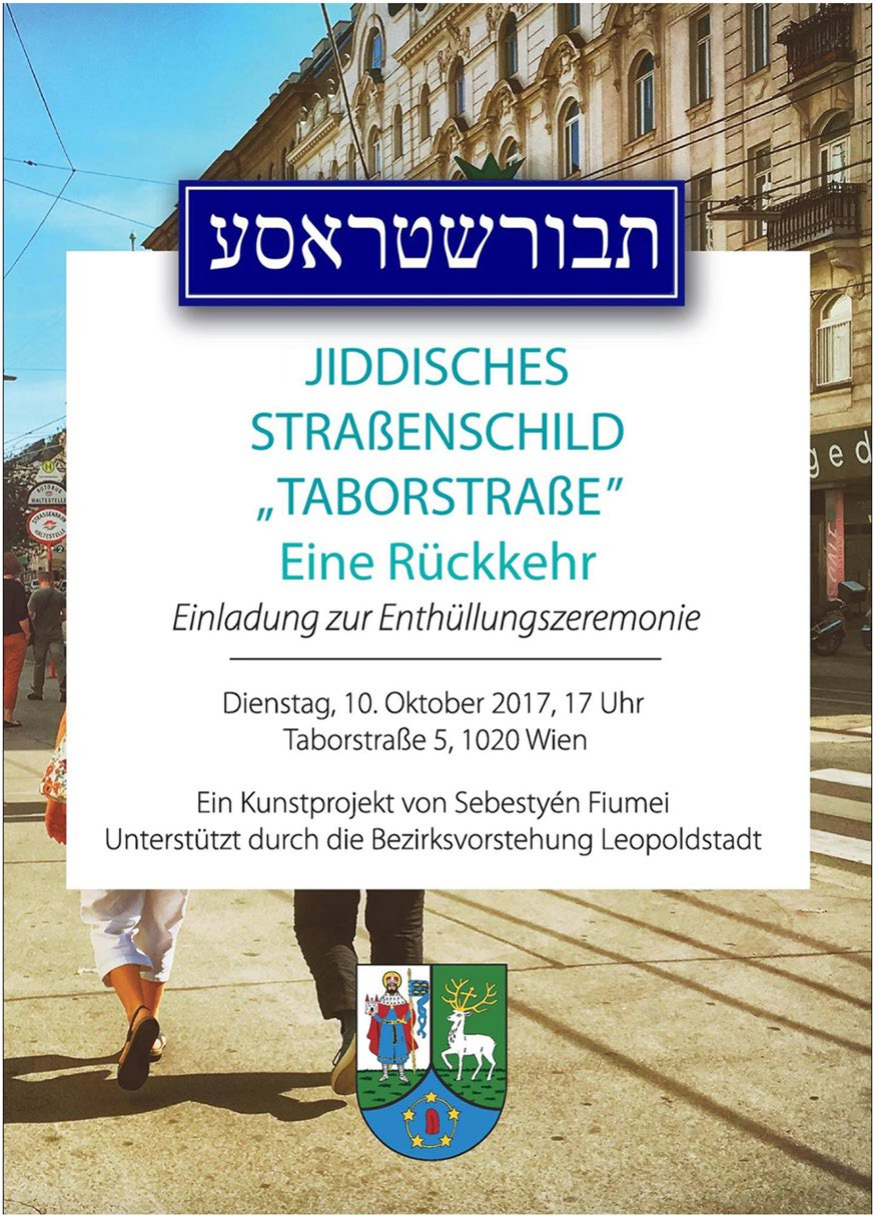
Flyer “invitation to the revival of the street sign”

Following a few brief newspaper article and some weeks of activism on the Internet, the virtual community finally gained attention in communal politics. After contacting both the local community and the artist, politicians Andrea Standl and Adi Hash, both members of the Green Party, initiated the re-erection of the street sign and called for an event that would bring it back to the *Taborstraße* neighborhood. Supported by Ursula Lichtenegger (Green Party), who operated as head of the *Bezirksvorstehung Leopoldstadt* (district administration) at the time, the reinstallation was framed as the opening of an art installation by Fiumei in an “Enthüllungszeremonie” (unveiling ceremony) on 10 October 2017 (Fig. 2). It seems like a healthy way to politically stage the importance of Jewish cultural heritage for the neighborhood—a staging that, if one believes newspaper coverage, could cynically unfold its performance even more strongly through a right-wing politician.

The official event was widely followed, and members of Jewish and non-Jewish communities of Vienna participated. For instance, the president of the Viennese Jewish Congregation Oskar Deutsch, vice presidents Chanan Babacsayv and Dezoni Dawaraschwili, and chief rabbi Arie Folger, as well as politicians, contributed to the official unveiling. Local newspapers reported on the event, and the reinstallation was a great success.^4^ The Jewish artist presented the sign to the district administration and to Deutsch as the official representative of the Jewish community. Deutsch was quoted to have said to wish for such a street sign on every street in the second district (Bezirksblatt 2017a). Pleased, honored, and inspired by the commitment of the local community, the Jewish artist stated: “Sometimes it only takes one person to get a piece of art removed, but dozens of others to get it put back up. I think this case was a good example of that. Nevertheless, I was very happy about the support of the local Jewish communities of the second district” (Correspondence 2023). Yet, it is important to note that the street sign was placed at Taborstraße 5 instead of on the facade of Taborstraße 18, the former Hotel National (famous for hosting a vaudeville stage at the turn of the twentieth century), where it was initially installed. Taborstraße 18 belongs to the hospital of the Brothers of Mercy. When Fiumei claimed that he wished to re-place it at the building, he was informed that renovations would soon be made to the building and that he should return “when the building is ready for it” (Correspondence 2023). Asked for his reasons as to why he wanted to put up a street sign, the artist replied that he considers it a nice gesture when, for instance, “[…] a place name is placed not only in the official language of the country, but also in a language that concerns many inhabitants of the place with their culture[…],” and hinted at the well-known example of Chinatowns. Yet, as he admitted, “in Austria, on the other hand, such a thing is not always so welcome.^5^ […] By putting up a street sign, I also wanted to counteract the fact that, ironically, the most Jewish quarter in Vienna is named after an antisemite.” Indeed, the second district owes its name to Leopold I and his role in the expulsion of the Jewish population from the city in 1669/70.^6^ In debates about either renaming or contextualizing historically problematic streets, squares, etc., Fiumei considers himself as “not necessarily taking the position of the renaming proponents. I am more in favor of counterbalancing. I believe that we can learn more about history and society through this” and anticipates his art as a step toward critical counterbalancing spaces (Correspondence 2023). Yet, given the vivid discussion about appropriations of Jewish and Yiddish cultures by non-Jews, one may wonder whether the artist fully grasped the meaning or consequences of his intervention (Gruber 2009, 487–9).

To conceptualize the interplay between local engagement and the online community, I want to point to another debate on creation, naming, and artistic intervention in public spaces in Vienna: the antisemitic mayor Karl Lueger and his (still remaining) representation in the contemporary cityscape. Until 2012, a part of Vienna’s prominent *Ringstrasse* in the very center of the city (the part where the university is located) was named after Lueger (Luegerring, today Universitätsring). And until today, a monument—though artistically conceptualized—secures the antisemite’s presence in the cityscape. Politicians and academics, but above all the general public—still present in the virtual sphere, yet operating primarily on other media back then—have campaigned for the street to be renamed and for the monument to be removed and/or replaced. The former happened in 2012, and the latter is still only happening in the form of a palimpsest-like inscription. As Dirk Rupnow states, the debate on the potential removal and replacement of this monument indicates that “in other countries, streets are renamed and statues removed while Austria remains steadfast” (2023, 145). While a detailed discussion of the context and the problems with the *Luegerring* and the *Lueger Denkmal* would reach too far here, it should be briefly pointed out that the online documentation of the whole initiative^7^ and discussion that emerged around the controversial monument indicate another dynamic than in the case of the Hebrew street sign: In the case of the monument, which has not been removed but only contextualized, a local, unknown initiative eventually sprayed it with “shame” to bring its problematic back into the public discourse. While the support by the online community helped the Hebrew street sign to be re-placed, the dynamic of the Lueger monument discussion indicated another direction of action—namely action in the physical space that then promotes a virtual community to join in (Teig 2023, 72–3).

**Fig. 3:**
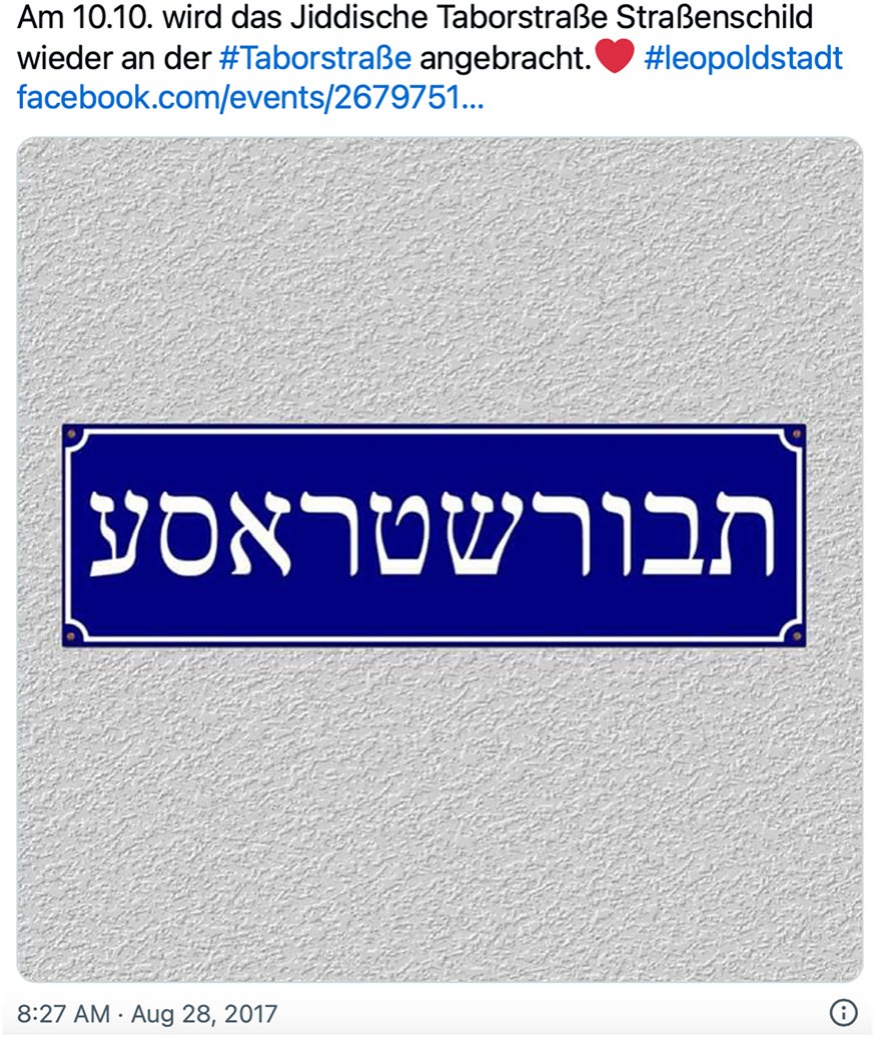
Screenshot by the author of extracted Tweets via https://tweet-entity-extractor.glitch.me/analyze

Since its reinstallation, the Hebrew street sign has become a trademark of Vienna’s second district, portrayed in many tourist pictures and widely seen as a representation of the neighborhood’s rich Jewish history. For example, X (formerly Twitter) application programming interface (Twitter API)^8^ data can be used to track how often the street sign, or an image of it, is used as a reference to the second district, its Jewish history, and the current urban atmosphere that surrounds it. Drawing on the data that Twitter users choose to share publicly with the world highlights an increase in references to Jewish history and culture in the second district related to the placement, removal, and finally re-placement of the Hebrew street sign (Fig. 3).^9^ What is more, the virtual presence initiated another discussion on the Internet concerning the topography of Tabor Streets across the globe. The virtual community learned that there are Tabor Street signs in Jerusalem and Brooklyn, New York (Facebook Group Taborstraßenschild 2023). Another example of the attention the sign gained is a video used for urban communication design in which a group of students at Technical University Vienna placed the Hebrew street sign, then in an abstract and digitalized visualization, at the heart of a video clip on typography in Vienna.^10^

Fiumei also received reactions of both Jewish and non-Jewish residents and of diverse Viennese Austrians. The artist recalled that some “native Austrians” were annoyed by the street sign: “They thought it was foreign because it is not in German. But this is wrong, and this is also what this project wants to demonstrate. Jewish history is not foreign, certainly not in the second district. It belongs to this district; it belongs to Vienna. It is also absurd to believe that the German language is threatened by Yiddish or Hebrew” (Correspondence 2023). Despite these backward positions, the artist was happy to see support and appreciation for the street sign, especially by the heterogeneous Jewish communities that were present: “And it was not only the Ashkenazi Jews who thought the project was important; through my former partner, who is from the Caucasian Jewish community of Vienna, I learned to my surprise and delight that the project was also much discussed and appreciated by Caucasian, Bukharian and Georgian Jewish women in Misrachi Jewish hair salons, for example. And, of course, the recladding was supported by many non-Jewish residents of the neighborhood” (Correspondence 2023).

## The Digital Culture, the Spatial Turn, and a Virtual Sphere in Jewish Studies

“The” Jewish space of Europe has become a widely discussed issue. Diana Pinto refers to questions of contemporary Jewish space making when arguing that “the Jewish Space [in Europe] constitutes a new phenomenon which marks a sea change in the map of Europe’s own consciousness and identity” (Pinto 2022, 180). Indicating the present popularization of Jews, Jewish themes, Jewish cultures, and Jewish life, Pinto argues that the last two decades have witnessed an ever-increasing re-presence of “the” Jewish space in Europe. Yet, as Pinto along with others have demonstrated clearly, this does neither spell out antisemitism nor prevent philosemitic attempts. (Gruber 1996, 1; Pinto 2022, 178–9). The Jewish

includes everything from Jewish websites to debates about the incorporation of Jews and Jewish life into national identity, from Gentile klezmer bands to memorial name-readings of Shoah victims, from inter-faith dialogue to Israeli folk dancing courses, from Jewish Studies programmes at universities to bagels. […] It is not populated solely by Jews; indeed, it may exist even without any Jews at all (Leveson and Lustig 2022, 188).

Keeping the space making through a Hebrew street sign in mind, I wish to point to Ian Leveson and Sandra Lustig’s notion in their definition of “the Jewish space of Europe,” namely, a wide range of activities, people, cultural actions, educational interests, memory-related initiatives, and last but not least, the virtual sphere that today shape “the” European Jewish space—or rather European Jewish spaces, if I may say so.

In the past decades, Judaism and Jewish culture have also been increasingly negotiated, lived, and constructed on the Internet. It is not only that cultural and religious institutions and heritage sites offer activities, participation, and information via online channels.^11^ Using the case of a virtual pop-up community, Nathan Abrams, Sally Baker, and B. J. Brown demonstrate that people—Jewish and non-Jewish alike—gather on the Internet and form religious and denominational communities, exchange about religious practice, discuss exegesis, make music together, read historic and contemporary texts, negotiate cultural installations, and add much more to Jewish culture and life (2013, 145; Abrams 2015). Peter Margolis highlights that the Internet functions as an amplifier of the lived world and offers many possibilities of cultural and religious actions to be added to Jewish life (2023, 189–92). Since the 1980s, the Internet has thus become a space for encounters, both literally and metaphorically, and it also provides the necessary infrastructure to create Jewish spaces in the physical world.

Since notions of spatiality have vividly resonated with postmodern mores in the field of Jewish studies, I wish to introduce some of its premises to learn about the entanglement of digital culture and spatial design. Dating back to the 1990s, research has increasingly become interested in the composition, construction, constitution, and making of Jewish places and Jewish spaces, leading to the current heyday of a Jewish spatial turn. Throughout the more than three decades since, investigations of Jewish spatiality have developed in multifarious directions, including religious sites, urban Jewish landscapes, (critical) mappings of memorized Jewish spaces, and interest in (historic) spaces of encounters and spheres of everyday life. Researchers from diverse disciplines, such as religious studies, urban histories, and cultural studies, have expressed interest in heterogeneous Jewish spaces (Brauch et al. 2010, 19–22; Mann 2012, 2–3). Alongside these varied research interests, a vast number of definitions and approaches to Jewish places and Jewish spaces have developed. Hitherto, they have been discussed broadly, sometimes even controversially or contradictorily (Korbel et al. 2020, (2). For instance, Charlotte Elisheva Fonrobert and Vered Shemtov investigated Jewish spaces through the lens of their constitutions in written text (2005, 2). Arijit Sen and Lisa Silverman understood spaces as containers for place making—a process that endows spaces with meaning (2014), while Julia Brauch, Anna Lipphardt, and Alexandra Nocke (2008, 4) regarded Jewish places as everything that has a physical position in a geographical landscape and a defined Jewish affiliation.

Despite the increasing influence of the Internet, the virtual world and digital culture—for negotiations of Judaism and Jewish history and culture as well—and the omnipresence of the metaphor of “digital space,” spatial considerations have so far received little attention in the so-called spatial turn (Campbell 2015a, 2015b, 5–7). For example, Martina Löw and Gunter Weidenhaus emphasized that spatial references in the context of the scissors of the Internet often decouple debates about reciprocal spatial design into the sheer metaphorical level. Yet, the increasing presence of the virtual aspects or the way virtual negotiations might condense in physical spaces has received less attention (Löw and Weidenhaus 2017, 553–70). Besides approaches inspired by digital humanities, few works have been interested in Jewish spaces and virtuality (Knowls and Rossetto 2022).^12^ According to the historians Miriam Rürup and Simone Lässig, questions regarding relational spaces—spaces as products of social interactions and sociability—are still underrepresented, and in particular, ignored in terms of the virtual world (2017; Zsívós 2015).^13^

When Rürup and Lässig edited the anthology *Space and Spatiality in Modern German-Jewish History*, they were among the first to open the spatial turn in Jewish studies toward the digital sphere. In their reflections on what the Jewish spatial turn has achieved hitherto, they included, for instance, Ruth Ellen Gruber’s reflections on her concept of “virtually Jewish”—a punning reference to the Internet that rather mediates the multifarious understandings of space making and its negotiations, in, beyond, and in-between physical, imagined, virtual, and/or memorized spaces. It is important to note, though, that with the widely discussed concept “virtually Jewish,” Gruber does not have the virtual world in mind but suggests that “in post-Holocaust places already now devoid or nearly devoid of living Jews, non-Jewish interest in Jewishness has had this effect,” namely, to produce a “virtually Jewish world” (2017a, 300). Works such as those by Barbara Mann indicate that the virtual sphere has also started to add to physical Jewish spaces, and other approaches have demonstrated that in the virtual sphere, Jewish spaces might evolve as, for example, Joachim Schlör examines with the democratic preservation initiated by a Facebook group using social media as an archival space (Schlör 2023, 145–7). Finally, the dynamic between real-life spaces and those created on or through the Internet resonated in Maja Hultman and Joachim Schlör’s (2021) call for a 2022 conference on “Jewish spaces in past and present Europe,” during which they aimed to discuss contemporary Jewish spaces as both “virtual and real-life spaces.”^14^ So, will “new Jewish spaces” be continuously initiated in the virtual sphere (Pinto 2002, 250)?

## The Case of Vienna and Hebrew Street Signs in Other European Cities

As in Vienna, Hebrew (and Yiddish) street signs are increasingly common in European cities. Especially in the post-socialist countries, such street signs belong to the phenomenon Gruber described as “virtual Jewishness.” The story behind the Viennese street sign is rather different; debates surrounding it demonstrate the wide range “virtually Jewishness” takes, beyond Eastern Europe and post-socialist countries as well. Before I aim at contextualizing what I consider practices of space making, I wish to introduce Fiumei’s aim and thoughts regarding the Viennese Hebrew street sign and this particular kind of art installation.

Interested in the multilayered processes of spatiality that unfolded alongside the Viennese Hebrew street sign, I was eager to talk about them with the artist. In correspondence with him, I learned that Fiumei specializes in working with languages and shares an interest in urban spaces, especially in what he called the “socio-history/ies” of certain neighborhoods. He shared with me that one of the places he lives is the second district of Vienna (which he actually prefers not to refer to as *Leopoldstadt*):

Taborstrasse is the main artery of the so-called Mazzesinsel, there is no other street in Vienna that is so closely and intensely connected with the Jewish past, but also with the Jewish present. I was intrigued by the coincidence that the word Tabor also happens to resonate with the religious Jewish residents of the neighborhood. The term Tabor is a wartime loanword that entered the German vocabulary during the Hussite period. Originally, Tabor was the name given by the Taborites, the radical and militant wing of the Hussites, to a place in the open air where they gathered, a camp. The exaltation of Jesus on an unnamed ‘Mount of Transfiguration’ recounted in Mt 17:1-12 EU was placed by the Taborites (as were other Christian groups) on Mount Tabor. Mount Tabor (in Hebrew: Har Tavor) and its surroundings play a central role also in the Tanakh in the Book of Judges, chapter 4 ‘Deborah Battle’ (Correspondence 19 June 2023).

Being aware that Internet campaigns are not automatically positive, this collaborative construction of a Jewish space in mutual exchange between a virtual community, local activists, and regional politicians in Vienna’s second district ultimately enabled an art intervention in the public space in Vienna that soon had an impact beyond the city’s borders. Following the Vienna Hebrew street sign in 2017, Sebestyén Fiumei designed similar signs for installations in Paris and Berlin. As had been the case in Vienna, his signs evoked a range of reactions from the local communities and politicians. All three projects place a Hebrew or Yiddish street sign in surroundings strongly associated with Jewish culture, whether historically or at present. “I think there are quite a few monuments and other objects that remind us of the terrible things that happened in Jewish history, but sometimes I miss things that would remind us of the actual life and stories and people between these tragedies” (Correspondence 19 June 2023). A crucial part of his art activism is the moment of surprise, as Fiume told me: “I did all three installations in public spaces without prior permission, this is part of my artistic practice, I want to keep the element of surprise, similar to street art in general, just maybe overcome the vandalism” (Correspondence 19 June 2023).

In Paris, Fiumei created a Yiddish street sign that reads “Pletzl,” which is a reference to the Jewish quarter of the fourth arrondissement in Paris. Comparable to Vienna’s *Taborstraße*, Paris’ *Rue de Rosiers* holds a specific place in the representation of historic, memorized, and present Jewish culture. The street is known for serving as a space of Jewish and non-Jewish encounters, past and present, as well as being a hub for heterogeneous Jewish communities, including Hasidic and orthodox Jews and liberal groups with diverse migration backgrounds from Eastern Europe as well as from North Africa (Brody 1996, 357). Fiumei placed the sign at the heart of *Rue de Rosiers* in 2019 (Plaque 2019). “In the Parisian Jewish neighborhood of Pletzl, my project was immediately understood and liked, even the mayor of the arrondissement posted a picture of it on his Instagram profile. It now acts as one of the attractions of the neighborhood, you can even buy fridge magnets of it in the local Judaica stores” (Correspondence 19 June 2023).

**Fig. 4:**
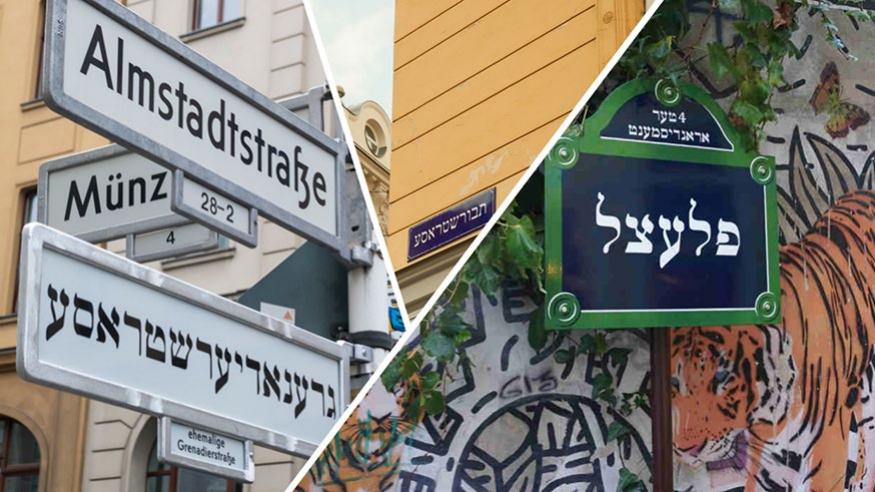
Collage of Fiumei’s projects in Vienna, Paris, and Berlin (by the author)

Following the Paris 2019 project, Fiumei began work on a Yiddish street sign for Berlin’s *Grenadierstraße* in the center of the former *Scheunenviertel* (barn quarter), the port of call for Jewish immigrants from Eastern Europe and the Russian Pale of Settlement in the first decades of the twentieth century. Similar to Vienna’s *Leopoldstadt* and Paris’ Pletzl, in the *Scheunenviertel* a rich Jewish culture was represented, as it was a sphere where orthodox Jews, new immigrants to the city, and those climbing the social ladder mingled and where the most prominent spots of Jewish religious and cultural life were found (Saß 2017). In a newspaper interview, Fiumei stressed that despite Berlin’s abundance of memory culture, one is astonished to find almost no hint of the Jewish history of this street and quarter (Malburg 2021). With this project, Fiumei aimed to do what he called “memory culture from below.” As was the case with the two other installations, he again consciously evaded bureaucracy when he placed his art at today’s *Almstadtstraße*, which was called *Grenadierstraße* in earlier days (Yiddish Berlin 2021). Yet, “[i]n Berlin it was removed by the authorities, but the neighborhood assured me that they would be interested in installing it.” However, due to regulations for artwork in public spaces in Germany that forbid artwork to be placed only temporarily in public spaces, Fiumei is still trying to bring his sign back to the street. So far, the sign has been kept in storage by the city administration (Fig. 4).

With the installation of the street signs by Fiumei in the three cities, two aspects of negotiation of contemporary Jewish spaces became evident. On the one hand, discourses around visible Hebrew font and Yiddish culture in urban space overlap. On the other hand, today’s Jewish space making in Europe is intrinsically linked to memory. What is particularly compelling about the Vienna art installation is that the sign is usually referred to as a Yiddish street sign and not a Hebrew one; however, a reader of the languages will immediately notice that it is not a Yiddish transliteration of the German street name. In addition, one of the online debates, reflected in comments in the Facebook group, revolved around the fact that the supposed Yiddish transliteration of the street name would be incorrect.^15^ And yet, the artist’s point was precisely not to transliterate a given name but to add an explicit biblical reference to Mount Tabor: “It was a conscious decision not to simply transliterate the word Tabor into Yiddish (טאבאר), but to use the spelling of the biblical mountain (תבור). I found it a happy coincidence that the name of the street also has an indirect Jewish reference. I wanted to create an association with Mount Tabor. I wanted to point to that association, to reinforce it” (Correspondence 19 June 2023).

## Virtual Jewish Space Making

But how does the street sign initiative fit among and contribute to virtual Viennese Jewish spaces? Vienna, alongside other cities, is obviously the subject of a broad discourse on the Internet today. The hashtag *#JewishVienna*,^16^ for example, collects a wide variety of contributions related to research institutions: in particular, the Vienna Wiesenthal Institute and the Jewish Museum, memorial initiatives, such as *Politics of Remembrance* (POREM), or a project on an apartment building at the *Servitengasse* and its expelled Jewish residents, and private groups that exchange information about former relatives who once lived in Vienna but had to flee or were murdered. The hashtag *#JewishVienna* might also be attributed to posts and contributions by individuals on social media and thus adds to the portrayal of the Jewish spatial perception of the city. Despite such loose affiliations that can easily be added to link interests, I am interested in a more specific form of exchange, influence, and transgression between the virtual and physical space with this case study: namely, how a mutual back and forth between online and physical spaces fosters sociability. In the case of the *Taborstraße* street sign, a real-life and a virtual community together generated an impact on a public space through Facebook and other social media activities that raised public awareness and put pressure on the city administration. First, the installation of the street sign can be described as a relational spatial practice that allows for a discussion of how an abstract spatial conception by an informal, non-group-based, non-governmental initiative operating on the Internet took shape in a physical location. Triggered by the removal of the sign, a community popped up using (primarily) Facebook to operate and form, before transgressing their action into the physical space; an art project recreated the Hebrew street sign *Taborstraße*, which was then re-established on 10 October 2017. Finally, as the sign was placed in the streets of Vienna again, it again impacted discussion and cultural negotiations on the Internet and soon led to artistic installations elsewhere that were meant to provoke public reactions to a visible, allegedly officialized lived Jewish culture as amalgamated in city administrations’ naming of streets.

The debate around the removal and reinstallation of the Hebrew street sign in Vienna was a beginning rather than an end of a hybrid way of constructing Jewish urban spaces. Since then, an even more vivid exchange between virtual and physical activities has shaped perception of the city’s Jewish spaces, so to speak. In terms of the discussion of Yiddish culture in the public sphere created in a historic (but also contemporary) Jewish space by (also) non-Jews, Vienna’s *Taborstraße* street sign would perfectly fit into examples of “virtual Jewishness.” As I aimed to demonstrate with my examination of the intermingling of pop-up virtual and real-life communities, the space-making practice emerging around the *Taborstraße* street sign is an example of a collaboration between a virtual and a local community in which Jews and non-Jews strongly interacted. This dialogue added new layers to the historic Jewish quarter of Vienna and to how people interact in, come into contact with, and perceive the *Leopoldstadt*.

This Viennese example differs from conventional phenomena of space making insofar as it did not (only) remain at the level of “virtually” or invention but instead enhanced a mutual and dynamic ongoing exchange between real-life and virtual spaces. The pop-up community-initiated neighborhood activism was in answer to a political debate, first on the Internet and then in the physical space, and then resonated among a larger global online community. Through its online presence, the group was also able to launch a global search for similar installations and created a huge *Taborstraße* street sign network. This connected diverse people who started to exchange, think, and thus recreate the atmosphere of their district and personal and family memories related to it, to Vienna, or even more generally. They discussed findings and space making in Europe and around the globe, while *klezmer* festivals are increasingly being set up in Krakow’s Kazimierz or in Vilnius and other Eastern European cities (Waligorska 2013) as virtual and/or “virtually” initiatives and evoke sociability and Jewish-non-Jewish relations, for instance among festival visitors. Additionally, touristic sociability around “virtually” and virtual created spaces is more and more paradigmatic for today’s Europe as it witnesses the heyday of “Holocaust tourism” (Gruber 2009, 498–500). But the installation of a street sign in Vienna might be added to what Gruber describes as a dynamic transition from “virtually Jewish” (Gruber 2009, 2017)—which, as mentioned, describes the invention of European Jewish spaces by non-Jews—to the virtual; a phenomenon we are, according to Gruber, currently intensively witnessing.

## Conclusion: Spaces Meet Digital Formats—Spatial Theory as a Hybrid Undertaking

The Viennese initiative is but one example of a change in Jewish space making—one that also/and/or may include the virtual sphere and online communities. In this article, I investigated Jewish space making as a dialectic and mutual dynamic between real-life spaces and the virtual space. The discussion concerning the *Taborstraße* street sign and the ceremony for its reinstallation introduced a scenario in which Jews and non-Jews alike participated in an online campaign. Despite all the problems that Internet campaigning brings, and all the many (and not fully graspable) intentions behind such art installations, the Hebrew street sign should be considered one example of new hybrid undertakings in space making and including the virtual sphere (not in Gruber’s sense of virtually Jewishness). I argue that such examples should be considered increasingly meaningful components of contemporary space making, adding to “the” Jewish Space in Europe by contributing manifold and heterogeneous Jewish spaces. Case studies such as the *Taborstraßenschild* initiative and its impact on the making of Jewish space in other European cities reveal new hybrid relational formats of Jewish spatiality in modern Europe, despite the long-standing ban of virtual spaces in the spatial turn. This late inclusion of virtual spaces was not least due to the strict rejection of their inclusion in relational concepts by the concept’s founder, Martina Löw, who argued that the Internet is the implication of a spatial metaphor—Löw and Weidenhaus define it as diametrically opposed to spaces: “In short, the Internet is overall not a space, but rather a communication system that embeds spatial metaphors” (2017, 556). Regarding consideration of Jewish spatiality, there has been a significant presence in cyberspace for the past decades, and channels of virtual communication have changed a lot since. It has thus become increasingly apparent that there are transitional scenarios of physical space making. Virtual spatial constellations are anchored in physical places and also anchor themselves in and through social relationships, which then in turn affect and impact the negotiation in virtual spaces (on the Internet in general, in distinct social media in particular). This becomes apparent when one moves through the streets of Vienna’s second district—virtually or physically—and witnesses how people arrange to meet at the Hebrew-letter *Taborstraße* sign or come into contact and exchange ideas through the initiative, even globally via the city borders dialogue, which then catalyzes concrete meetings in Vienna. We are thus also seeing, in Diana Pinto’s sense, new Jewish spaces as hybrid Jewish spaces (2002, 242–50)—and hybridity in an additional sense as it was described in the postcolonial turn (Bhabha 2004). Bringing spatial approaches and humanities methods together adds new possibilities for analyzing contemporary Jewish virtual and real-life spaces. And if I may add a wish for future investigations: data-driven sociological analysis could provide further illumination and should be conducted to more adequately understand relational space making as, for example, around the Hebrew *Taborstraße* street sign initiative.

## Footnotes

1 Das Taborstraße-Schild auf Jiddisch soll zurück, https://www.facebook.com/profile.php?id=100067922265410. Accessed 14 November 2023.

2 Taborstraße Straßenschild/תבורשטראסע שלט רחוב, https://www.facebook.com/Taborshtrase-. Accessed 14 November 2023.

3 The street artist Sebestyén Fiumei is well known for urban art installations that relate Jewish history to cities’ present appearances. For instance, Fiumei also made the sign “Mazzesinsel” in Yiddish letters at Schwedenbrücke in Vienna and Yiddish street signs at Rue de Rosiers in Paris. Recently, Fiumei developed a street sign for Grenadierstraße in Berlin (Facebook profile: https://www.facebook.com/shabi.fiumei/; Twitter account: https://twitter.com/shabi_fiumei).

4 There was also a minor scandal because a politician of the right-wing Freedom party participated and tried to claim the installation as supported by them (Taborstraße 2017).

5 Fiumei here hints at the vandalism of the bilingual place-name sign of Oberwart, where the Hungarian name Felsőőr was scratched through, and the dispute about bilingual place-name signs in Carinthia.

6 On the *zweite Wiener Gesera*, see Staudinger 2005.

7 https://www.luegerplatz.com Accessed 10 November 2023.

8 Twitter API provides programmatic access to data that was publicly shared on Twitter, such as images, hashtags, and written posts about the Hebrew street sign in Vienna. Twitter API helps to “analyze, learn from, and interact with Tweets, Direct Messages, and users.” It can be used to, for example, investigate an increase or decrease of mentions, references, comments, and other tweets related to the street sign (see https://help.twitter.com/en/rules-and-policies/twitter-api). For more on the advantages and limitations of such an analysis, see Dongo et al. 2020.

9 Data collection and analysis based on my open access Twitter API account: API20172023NN.

10 https://www.youtube.com/watch?v=UlbtwoDxHPo

11 Ranging everything from the Western Wall to congregations around the world to music festivals to literature and to archives, museums, libraries, special collections, etc., the online offerings for participation in Jewish religion and culture have grown immeasurably with the rise of the Internet (Campbell and Fulton 2013, 185–90).

12 In particular, research on the Holocaust has resonated with digital humanities approaches and digital as well as critical mapping. See, for example, Anne Kelly Knowls’ *Holocaustgeographies*; on deep mapping in Jewish migration history from the Middle East and North Africa, see Rossetto 2022.

13 What is more, Lässig and Rürup highlighted that historical research in Jewish studies has ignored questions concerning spaces. Among the first historical studies of Jewish spaces was a special issue of *East Central Europe* (vol. 42, 2015).

14 The call can be accessed via the website of the European Association for Jewish Studies, https://www.eurojewishstudies.org/conference-grant-programme-reports/report-a-jewish-europe-virtual-and-real-life-spaces-in-the-21st-century/. Accessed 10 November 2023.

15 For example, user Michael Krebs (15 September 2021) stated, “Meiner Meinung nach ist das kein Jiddisch, auf Jiddisch würde es wohl תבורגאס (Taborgasse) lauten.” Facebook Group Taborstraßenschild. https://www.facebook.com/permalink.php?story_fbid=pfbid02bZqYR4MBMJhKHvqKwnsPYqVJzQMcqecCf3vZr2R7mYr87dhbeCcaCY7YwpKAm38cl&id=176065299601142&comment_id=1073665409841122. Accessed 24 June 2023.

16 Vienna Wisenthal Institute, https://www.vwi.ac.at; POREM Politics of Remembrance, https://porem.univie.ac.at; Servitengasse Verein, https://www.servitengasse1938.at/index.php?id=2; Jewish Museum Vienna, https://www.jmw.at. All accessed 24 June 2023.
